# Editorial: The role of neuromodulation techniques in facilitating motor recovery and brain plasticity: an integrative approach

**DOI:** 10.3389/fnhum.2025.1620300

**Published:** 2025-07-15

**Authors:** Egas Caparelli-Dáquer

**Affiliations:** Laboratório de Estimulação Elétrica do Sistema Nervoso - LabEEL, CePeM - HUPE, Universidade do Estado do Rio de Janeiro - UERJ, Rio de Janeiro, Brazil

**Keywords:** neuromodulation, TMS—motor-evoked potentials, repetitive transcranial magnetic stimulation (TMS), neurorehabilitation, neuroplasticity, transcranial electric stimulation, vagus nerve stimulation (VNS), noninvasive brain stimulation (NIBS)

Motor disabilities can lead to long-term impairment, dependence, and reduced quality of life, representing an enormous burden for patients. This reality affects not only older adults (Herbert et al., 2020) but also children (Tsai et al., 2018). A recent study from India reports an overall disability prevalence of 4.52%, with locomotor disabilities being the most common (Pattnaik et al., 2023).

Despite this pressing need, available therapeutic options often demand toil, tears, and sweat—sometimes even blood—yet frequently yield results that fall short of expectations, particularly when contextual factors do not support active engagement in rehabilitation strategies. Anyhow, on average, improvements following traditional rehabilitative therapy are moderate. A recent Cochrane review on physical rehabilitation for stroke suggests that rehabilitation improves motor function with a standardized mean difference of 1.01 (95% CI: 0.80–1.22) compared to no rehabilitation (Pollock et al., 2025).

This scenario calls for techniques to augment existing rehabilitation efforts. Neuromodulation techniques are increasingly recognized for their role in facilitating motor recovery and promoting brain plasticity, especially following neurological damage. Non-invasive methods, such as transcranial magnetic stimulation (TMS) and transcranial electric stimulation (TES), can modulate cortical excitability and induce plastic changes in the brain, through diverse mechanisms, including synaptic effects as well as structural plasticity, facilitating recovery by improving learning, memory, and motor functions, with therapeutic implications for motor recovery (Barbati et al., 2022). These techniques have been used alone or in combination with other traditional neurorehabilitation therapies to enhance neuroplasticity and improve functional recovery, allowing them to be integrated into rehabilitation programs to optimize recovery outcomes (Eliason et al., 2024; Liew et al., 2014).

This Research Topic in Frontiers in Human Neuroscience includes four articles on neurorehabilitation using neuromodulation tools for treatment or diagnostic purposes.

The article by Kumar et al. from Moss Rehabilitation Research institute uses TMS to assess corticospinal tract integrity after stroke. Their innovative approach evaluates intersegmental coordination during motor tasks involving proximal and distal muscles, offering a potential prognostic tool for stroke recovery.

Arias and Buneo from Arizona State University investigate trigeminal nerve stimulation (TNS) as a modulator of motor learning in an upper extremity visuomotor adaptation paradigm. Their findings reveal frequency- and timing-dependent effects of TNS on learning, suggesting that optimizing stimulation parameters could enhance rehabilitation outcomes.

The article of Shen et al. reviews the use of motor imagery therapy in upper limb rehabilitation for stroke patients in China, discussing mechanisms, applications, and the challenges of integrating motor imagery into routine clinical practice.

The original article from Le Cong et al. from Niigata University, Japan studies the contribution of repetitive somatosensory stimulation (RSS) on skill acquisition and retention in sensorimotor adaptation. The study tests the hypothesis that whole-hand water flow (WF) as a paradigm of RSS, induces M1 disinhibition, thus enhancing motor memory retention. In this study TMS is employed to probe motor disinhibition. Results indicated that while WF reduced intracortical inhibition, it did not significantly affect skill acquisition or memory retention, providing valuable insights into the complexities of sensorimotor integration.

Collectively, these contributions highlight the different ways in which neuromodulatory techniques can influence research as well as therapeutic options to improve motor recovery, not to mention the growing importance of these technologies in the issue of neuroplasticity and rehabilitation.
